# Assessing the use of supplementary materials to improve genomic variant discovery

**DOI:** 10.1093/database/baad017

**Published:** 2023-03-31

**Authors:** Emilie Pasche, Anaïs Mottaz, Julien Gobeill, Pierre-André Michel, Déborah Caucheteur, Nona Naderi, Patrick Ruch

**Affiliations:** BiTeM Group, Information Sciences, HES-SO/HEG Geneva, rue de la Tambourine 17, Geneva, Carouge 1227, Switzerland; SIB Text Mining Group, Swiss Institute of Bioinformatics, rue de la Tambourine 17, Geneva, Carouge 1227, Switzerland; BiTeM Group, Information Sciences, HES-SO/HEG Geneva, rue de la Tambourine 17, Geneva, Carouge 1227, Switzerland; SIB Text Mining Group, Swiss Institute of Bioinformatics, rue de la Tambourine 17, Geneva, Carouge 1227, Switzerland; BiTeM Group, Information Sciences, HES-SO/HEG Geneva, rue de la Tambourine 17, Geneva, Carouge 1227, Switzerland; SIB Text Mining Group, Swiss Institute of Bioinformatics, rue de la Tambourine 17, Geneva, Carouge 1227, Switzerland; BiTeM Group, Information Sciences, HES-SO/HEG Geneva, rue de la Tambourine 17, Geneva, Carouge 1227, Switzerland; SIB Text Mining Group, Swiss Institute of Bioinformatics, rue de la Tambourine 17, Geneva, Carouge 1227, Switzerland; BiTeM Group, Information Sciences, HES-SO/HEG Geneva, rue de la Tambourine 17, Geneva, Carouge 1227, Switzerland; SIB Text Mining Group, Swiss Institute of Bioinformatics, rue de la Tambourine 17, Geneva, Carouge 1227, Switzerland; BiTeM Group, Information Sciences, HES-SO/HEG Geneva, rue de la Tambourine 17, Geneva, Carouge 1227, Switzerland; SIB Text Mining Group, Swiss Institute of Bioinformatics, rue de la Tambourine 17, Geneva, Carouge 1227, Switzerland; BiTeM Group, Information Sciences, HES-SO/HEG Geneva, rue de la Tambourine 17, Geneva, Carouge 1227, Switzerland; SIB Text Mining Group, Swiss Institute of Bioinformatics, rue de la Tambourine 17, Geneva, Carouge 1227, Switzerland

## Abstract

**The curation of genomic variants requires collecting evidence not only in variant knowledge bases but also in the literature. However, some variants result in no match when searched in the scientific literature. Indeed, it has been reported that a significant subset of information related to genomic variants are not reported in the full text, but only in the supplementary materials associated with a publication. In the study, we present an evaluation of the use of supplementary data (SD) to improve the retrieval of relevant scientific publications for variant curation. Our experiments show that searching SD enables to significantly increase the volume of documents retrieved for a variant, thus reducing by ∼63% the number of variants for which no match is found in the scientific literature. SD thus represent a paramount source of information for curating variants of unknown significance and should receive more attention by global research infrastructures, which maintain literature search engines**.

**Database URL** https://www.expasy.org/resources/variomes

## Introduction

Precision medicine relies on the evaluation of the pathogenicity of the sequence variants for a given patient. The health-related evaluation of these variants is mostly based on the thorough reviewing of the literature, which makes the task both time-consuming and, especially, labor intensive. Genomic variant knowledge bases [i.e. COSMIC (1), OncoKB (2), ClinVar (3), UniProt (4), etc.] also gather evidence-based contents to interpret the clinical actionability of a variant; however, these resources are neither comprehensive nor up-to-date. Therefore, the screening of the literature (5) is of major importance for clinical practice guidelines.

To characterize sequence variants—most of them being variants of unknown significance—clinical experts rely on the scientific literature to synthetize evidence. Since the assessment of variant pathogenicity requires the combination of different types of evidence (6), such as prevalence in healthy and disease-specific populations, computational prediction and functional effect, each publication is likely of importance, which interestingly establishes that retrieval for personalized health is primarily a recall-oriented task.

Furthermore, Jimeno Yepes and Verspoor (7) reported that a significant subset of information related to genomic variants is reported neither in the abstracts nor in full texts of scholarly publications, but only in the supplementary materials associated with the publications (8). In agreement with FAIR best practices, it is indeed critical to make available and findable the source data used in published studies, which is another challenge here. In practice, the results derived from high-throughput studies cannot be incorporated within the body of an article but are rather shared as supplementary data (SD). Such a source of information, including tables (e.g. XLS and CSV) and images (e.g. TIFF and JPG), has unfortunately been relatively ignored by the information retrieval community. It is worth citing the bioCADDIE evaluation campaign (9, 10), which to our knowledge was the only shared task combining text and data search and which was unfortunately discontinued after the first year. According to professional surveys (11) and curation recommendations (12), biocuration workflows do include digesting SD contents. However, curated databases do not capture the precise provenance of the evidential statement they record, therefore it is difficult to quantify how often supplemental files are used. Furthermore, apart from Variomes, literature search engines (e.g. PubMed, LitVar/PubTator and Europe PMC) do not index SD contents. Altogether, we therefore argue that personalized health will remain wishful thinking unless the information retrieval community invests significantly into the field. In this study, we explore the foundations for such an effort by designing original search methods and services, applied to SD and evaluated thanks to an original benchmarking dataset.

## Materials and Methods

We conducted both recall-oriented and precision-oriented analyses of the impact of SD on the recall of Variomes (13), a search engine supporting the curation of genomic variants using the biomedical literature. In the literature, as well as in SD files attached to publications, variants are labeled in very diverse forms (i.e. there are dozens of expressions to represent a given variant, including expressions at the genomic, protein or transcript levels), and there is no universal terminology for describing variants. The Variomes search engine relies on SynVar (14), a dedicated variant expansion system to expand the query and retrieve documents mentioning the variant in any of its forms. The system is able to compute from a given single nucleotide polymorphism (SNP) all the different description formats, including protein, transcript and genome levels and syntactic variations as found in the literature. The queried variant does not need to exist in databases like Single Nucleotide Polymorphism Database or Catalogue of Somatic Mutations in Cancer (COSMIC).

**Figure 1.**
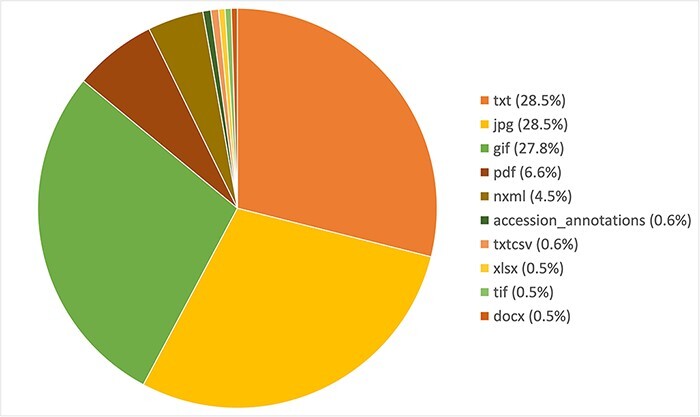
Distribution of the 10 most frequent file types in the Variomes supplementary data index.

Variomes is using three articles’ collections provided by the SIB Literature Services (SIBiLS) (15) and updated on a daily basis: MEDLINE abstracts, PubMed Central full texts and a collection of SD. This collection of SD contains a set of ~800 000 PubMed Central reference numbers (i.e. ∼20% of all PMC articles) based on the following equation: [(gene AND variant) OR polymorphism OR mutat*]. The files are basically of two types: text/tables and images. Tables are simply processed using text transformations (i.e. xls2txt), and images are OCRized with Tesseract (16). The resulting index contains 4 205 195 files, whose distribution per file type is shown in Figure 1.

### Recall-oriented analysis

The recall-oriented analysis relies on two benchmarks. The first benchmark (10.5281/zenodo.7661195) is oriented toward cancer mutations and consists of a subset of 803 genetic variants occurring in the genes *BRCA1* and *BRCA2* from the BRCA Exchange database (17). This subset corresponds to all reviewed single amino acid polymorphisms and nonsense SNPs from the 2017 LOVD dataset (18). The second benchmark (10.5281/zenodo.7661095) is based on ClinVar (3) mutations. It thus represents more diverse genetic variations. A random set of 1000 variants has been selected out of the subset of ClinVar SNPs resulting in a protein change.

These benchmarks are used to evaluate the impact of searching the literature with and without using SD. The implementation relies on three separate indexes for, respectively, abstracts (MEDLINE), full texts (PubMed Central) and the SD. For each variant of the benchmark, we retrieved documents from the three indexes using Variomes. Documents retrieved in MEDLINE and PubMed Central are used as a baseline to evaluate the impact of the SD. The impact is evaluated on two aspects. First, we evaluate the impact of SD to reduce the silence of Variomes. To this end, we first identify the set of variants returning no document in MEDLINE and PubMed Central. We then search for these variants in SD and count for how many variants we are able to identify at least one document in SD. Second, we evaluate how the SD enables us to retrieve documents that are not yet retrieved using MEDLINE and PubMed Central. We calculate the proportion of new documents returned in the SD compared to the documents retrieved in MEDLINE and PubMed Central.

### Precision-oriented analysis

To complement the recall-oriented analysis, we performed two analyses to assess the impact of SD on precision. First, we compared the proportions of the clinical significance levels—benign, pathogenic and unknown significance—of the variants found only in the SD with the variants from the rest of the benchmark. For this experiment, the BRCA and the ClinVar benchmarks are merged. Clinical significance information was retrieved from the respective databases of the benchmarks, BRCA Exchange and ClinVar. For clarity, all categories related to ‘benign’ mutations (e.g. benign and likely benign) were merged under the same ‘benign’ category, and all values related to pathogenic mutations (e.g. pathogenic and likely pathogenic) were merged into a ‘pathogenic’ label. All the other values (e.g. conflicting interpretations and not provided) were considered as ‘unknown significance’. Pearson’s chi-squared test of independence, using chi2_contingency from the Python package Scipy v1.9.3 (19), was performed to assess the difference in the frequency distribution of the clinical significance labels between the variants of both datasets. Second, we performed a manual screening on a subset of documents. We randomly selected two variants (i.e. *BRCA1*:R332Q and *BRCA1*:I1275V) from the BRCA benchmark and four variants (i.e. *BTK*:R520G, *MAP2K2*:D221N, *PMM2*:N216S, and *ANKH*:R187Q) from ClinVar to evaluate the relevance of the documents retrieved using the supplementary materials. We screened the first top 10 results to check the accuracy of the detected variant and the type of information found in the SD.

## Results

### Recall-oriented analysis

The recall-oriented analysis on the BRCA benchmark was performed on 1 March 2022, while the recall-oriented analysis on the ClinVar benchmark was performed on 4 March 2022.

The impact of SD to reduce the silence of Variomes is presented in Table 1. Using supplementary materials to search genetic variants enabled to strongly reduce the silence, i.e. the number of variant queries with no match in the article collection. About 63% of these ‘no match’ queries had a match in the supplementary material index. The impact was even higher for the BRCA benchmark: while 136 queries (16.94%) of the BRCA benchmark returned no result with MEDLINE and PubMed Central, only six queries had no match in the supplementary material index, thus resulting in a reduction of the silence by 95.56%. On the ClinVar benchmark, while a high number of queries (77.10%) were returning no document in MEDLINE and PubMed Central, using the supplementary materials enabled the retrieval of documents for more than half of these queries, thus reducing the silence by 57.20%. On average, 4.82 documents were retrieved for these queries in BRCA and 3.12 in ClinVar.

**Table 1.** Impact of the supplementary materials to retrieve documents for variants returning silence based on MEDLINE and PubMed Central

	BRCA	ClinVar	Total
Number of variants for which no documents were retrieved in MEDLINE and PubMed Central (baseline)	136	771	907
Number of variants from the baseline for which at least one document was retrieved in the SD	130	441	571
Average number of documents retrieved in the SD for these variants	4.82 (min: 1; max: 18)	3.12 (min: 1; max: 29)	3.51 (min: 1; max: 29)
Relative reduction of the silence when using the SD	−95.56%	−57.20%	−62.95%

The impact of supplementary materials to retrieve novel documents is shown in Table 2. For both benchmarks, a strong increase of the retrieved documents is observed. Indeed, on average, the use of the supplementary material index at least doubled the number of documents retrieved (on average +132.57%). In the BRCA benchmark, while we initially retrieved an average of 8.23 documents per variant using MEDLINE and PubMed Central, using the supplementary materials results in retrieving 9.64 new documents (i.e. +117.15%). In the ClinVar benchmark, an average of 1.26 documents were retrieved per variant in MEDLINE and PubMed Central, whereas using the supplementary materials enabled to retrieve on average 2.69 new documents (+213.59%).

**Table 2.** Impact of the supplementary materials to retrieve new articles compared to MEDLINE and PubMed Central

	BRCA	ClinVar	Total
Average number of documents retrieved in MEDLINE and PubMed Central (baseline)	8.23 (min: 0; max: 384)	1.26 (min: 0; max: 274)	4.36 (min: 0; max: 384)
Average number of documents retrieved in the SD	10.30 (min: 0; max: 74)	2.73 (min: 0; max: 94)	6.10 (min: 0; max: 94)
Average number of documents retrieved in the SD that are novel	9.64 (min: 0; max: 59)	2.69 (min: 0; max: 83)	5.78 (min: 0; max: 83)
Average number of documents retrieved in all collections (MEDLINE, PubMed Central and SD)	17.87 (min: 0; max: 440)	3.95 (min: 0; max: 357)	10.14 (min: 0; max: 440)
Relative gain of the SD compared to the baseline	+117.15%	+213.59%	132.57%

### Precision-oriented analysis

First, we broadly assessed the relevance of the information found in the supplementary materials retrieved for the queries of the benchmarks. We compared the clinical significance of the variants appearing only in the SD with the variants appearing in MEDLINE or PubMed Central (Table 3). While there was a difference in the distribution of the clinical significance values between both sets in the whole benchmark [X^2^(2, N = 1803) = 67.13, *P* < 0.01], the difference concerned mainly the proportion of variants of unknown significance, which was higher in the SD. The relative proportions of pathogenic and benign variants were not significantly different in the whole benchmark [X^2^(1, N = 583) = 0.09, *P* = 0.76] between variants found only in SD and the others.

**Table 3.** Frequency distribution of the clinical significance of the variants found only in the supplementary data compared to the variants retrieved in MEDLINE and PubMed Central

		BRCA	ClinVar	Total
Distribution of clinical significance for variants retrieved in MEDLINE and PubMed Central (*N = *1232)	Pathogenic	16.64%	19.14%	17.78%
	Benign	28.68%	11.09%	20.70%
	Unknown significance	54.68%	69.77%	61.53%
Distribution of clinical significance for variants retrieved in the SD but not in MEDLINE and PubMed Central (*N* = 571)	Pathogenic	7.69%	8.62%	8.41%
	Benign	3.08%	12.93%	10.68%
	Unknown significance	89.23%	78.46%	80.91%

We then evaluated the variants found in the supplementary documents for six queries, for a total of 20 documents in the BRCA benchmark and 21 documents in the ClinVar benchmark. We also tried to assess the type of information found about the variants (Table 4). The increased search effectiveness is consistent with the results found in the previous section: 100% of the SD were previously unseen documents for the two BRCA variants, while 95% were unseen for the four ClinVar variants. BRCA variants were found in more supplementary documents with on average 19.5 retrieved documents per query, compared to ClinVar variant requests that returned on average six documents. Two-thirds of the retrieved documents were correct, which provide an estimate of the precision of the search. When comparing the two benchmarks, we observed that the ClinVar benchmark showed less accurate results than the BRCA benchmark. Each supplementary document contained hundreds of variants. These results are not discussed individually in the full text either because they are benign variants appearing as part of an evaluation benchmark, because the evaluation does not demonstrate any pathogenicity or because they are discussed more generally in combination with other results. Regarding the type of information found in the SD, more than half were computational pathogenicity predictions, 22% reported allele frequency in various populations and 17% were genome-wide association studies (GWAS). Less informative data such as benchmark for computational prediction concerned assessed benign variants.

**Table 4.** Manual analysis of the documents retrieved in the supplementary materials

	BRCA	ClinVar	Total
Average percentage of novelty in the SD	100%	95%	98%
Average number (median) of documents retrieved in all collections (MEDLINE, PubMed Central and SD)	19.5 (min: 18; max: 21)	6 (min: 3; max: 8)	7.5 (min: 3; max: 21)
Average precision (median) for variants found in the SD	75% (min: 60%; max: 90%)	53% (min: 0%; max: 80%)	63% (min: 0; max: 90%)
Information type found in the SD	Pathogenicity prediction and allele frequency in population	Pathogenicity prediction, allele frequency in population and GWAS	Pathogenicity prediction, allele frequency in population and GWAS

## Discussion

The experiments reported in this paper constitute a first attempt to establish and quantify the importance of SD to support personalized health. Our results show that SD are a paramount source of contents to characterize the clinical actionability of sequence variants. However, some of the chosen experimental settings are likely to underestimate such a statement. Indeed, while the search for variants is benefiting from a quite powerful synonym generation engine, so-called SynVar (14), which can associate many synonyms of variants (e.g. *BRAF*:V600E, *BRAF*:Val600Glu, and *BRAF*:1799T>A), the variability of the gene (or gene product) names has not been similarly exploited. It means that the use of the synonyms of a gene or gene product (e.g. serine/threonine-protein kinase B-raf, *BRAF1*) could have further augmented the recall of our results; therefore, additional experiments would be needed.

While supplementary material represents an important source of information for curating variants, it also raises some challenges. First, supplementary documents often contain hundreds of variants from different genes, thus increasing the likelihood to match a variant with a wrong gene. Second, the processing of SD, and in particular content-based image recognition with optical character recognition (OCR), might generate some normalization errors (e.g. L wrongly recognized as £). Thus, and as reported by Wei *et al.* (20), some variants might simply not be recognized. Nevertheless, simple approaches or heuristics to improve precision could be implemented. For instance, it would be relatively straightforward to compute the positional distance (at word or offset level) between the gene and the variant. In parallel, improving precision should not be too detrimental for recall, especially with lesser studied variants for which very few publications exist. Ultimately, a user-piloted trade-off functionality between recall and precision could provide the flexibility needed to interactively switch focus on precision for the few highly studied variants, while being able to accommodate the need for broad recall for the overwhelming majority of sequenced variants.

## Conclusion

Supplementary materials associated with publications play a critical role in any literature curation pipeline (21), but this seems especially true for the curation of genetic variants. In our experiments, we identified that most of the documents retrieved through the supplementary material collection were simply not found when searching the full text of the articles. SD contents more than double the number of documents retrieved per query, thus significantly reducing the volume of variants for which no articles are identified in the literature. It represents valuable information for assessing rarely studied or unknown significance variant pathogenicity, including population studies or computational predictions. Finally, with a reduction of silence of 63%, our results are consistent—yet stronger—with previous observations by Jimeno Yepes and Verspoor (7), who reported that about half of the published content about genetic variations is found exclusively in the supplementary materials. While FAIR is becoming a top priority on the agenda of global research infrastructures (e.g. the US National Library of Medicine, the European ELIXIR community or the Global BioData Coalition), the proper FAIRification of SD should definitely receive more attention, in particular for research infrastructures maintaining literature search engines.

## Data Availability

The data used in this article are available under CC-BY 4.0 in Zenodo, at https://dx.doi.org/10.5281/zenodo.7661095 and https://dx.doi.org/10.5281/zenodo.7661195. The datasets were derived from sources in the public domain: ClinVar (https://www.ncbi.nlm.nih.gov/clinvar/) and LOVD (https://www.lovd.nl).
